# Structural Determinants for the Binding of Morphinan Agonists to the μ-Opioid Receptor

**DOI:** 10.1371/journal.pone.0135998

**Published:** 2015-08-17

**Authors:** Xiaojing Cong, Pablo Campomanes, Achim Kless, Inga Schapitz, Markus Wagener, Thomas Koch, Paolo Carloni

**Affiliations:** 1 Laboratory of Computational Biophysics, German Research School for Simulation Sciences GmbH, Joint venture of RWTH Aachen University and Forschungszentrum Jülich, 52425 Jülich, Germany; 2 Computational Biomedicine section (IAS-5), Institute of Advanced Simulation (IAS), Forschungszentrum Jülich, 52425 Jülich, Germany; 3 Computational Biomedicine section (INM-9), Institute of Neuroscience and Medicine (INM), Forschungszentrum Jülich, 52425 Jülich, Germany; 4 Grünenthal Innovation, Grünenthal GmbH, 52078 Aachen, Germany; Weizmann Institute of Science, ISRAEL

## Abstract

Atomistic descriptions of the μ-opioid receptor (μOR) noncovalently binding with two of its prototypical morphinan agonists, morphine (MOP) and hydromorphone (HMP), are investigated using molecular dynamics (MD) simulations. Subtle differences between the binding modes and hydration properties of MOP and HMP emerge from the calculations. Alchemical free energy perturbation calculations show qualitative agreement with *in vitro* experiments performed in this work: indeed, the binding free energy difference between MOP and HMP computed by forward and backward alchemical transformation is 1.2±1.1 and 0.8±0.8 kcal/mol, respectively, to be compared with 0.4±0.3 kcal/mol from experiment. Comparison with an MD simulation of μOR covalently bound with the antagonist β-funaltrexamine hints to agonist-induced conformational changes associated with an early event of the receptor’s activation: a shift of the transmembrane helix 6 relative to the transmembrane helix 3 and a consequent loss of the key R165-T279 interhelical hydrogen bond. This finding is consistent with a previous proposal suggesting that the R165-T279 hydrogen bond between these two helices indicates an inactive receptor conformation.

## Introduction

Opioid drugs, such as morphine, are widely used in clinics for the treatment of acute, postoperative, and chronic pain. Owing to their exceptional analgesic properties, they are consistently among the most commonly prescribed drugs nowadays [1]. However, although frequently highly effective, opioids consumption in a regular basis leads to the appearance of undesirable side effects, such as constipation or respiratory depression, which limit their clinical applicability. Moreover, their usage often leads to addiction, tolerance and withdrawal [2]. This poses a major problem for the use of the existing opioids in clinics, complicating dosing regimens for patients and strongly restricting the prescription of these drugs [3, 4].

Opioid compounds exert their analgesic and intense rewarding effects by acting upon opioid receptors expressed on the plasma membrane of neuronal cells. These receptors, as members of the class A seven transmembrane (TM) G-protein coupled receptors (GPCRs) superfamily, are specialized in transmitting stimuli from the extracellular environment to the cytoplasm [5]. According to their preferential interaction with endogenous opioid peptides, opioid receptors are divided in four different types: the nociceptin/orphanin FQ peptide receptor, and the classical μ-, δ- and κ-opioid receptors [6–8]. In spite of their relatively high sequence identity [9], they present a very specific functional outcome, each receptor being responsible for distinct pharmacological effects. In particular, μ-opioid receptors (µORs) in the peripheral and central nervous system mediate pain perception. They are the primary target of exogenous analgesics such as morphine and other prototypical opioid drugs [10]. Both beneficial and adverse pharmacodynamic effects of classical morphine-like drugs are attributable to the activation of μORs [11]. They may be separable by using biased ligands [12]. Indeed, it has been demonstrated in a large number of studies that different ligands, while acting on the same receptor, can stimulate and inhibit GPCRs signaling through several intracellular pathways differentially, not simply stimulating or inhibiting all pathways to the same extent. This phenomenon, termed functional selectivity or biased signaling [13, 14], is a key concept in GPCRs signaling, in general, and through opioid receptors, in particular. These receptors can signal selectively through G-protein and β-arrestin pathways in a ligand-specific manner [12, 15, 16].

**Fig 1:**
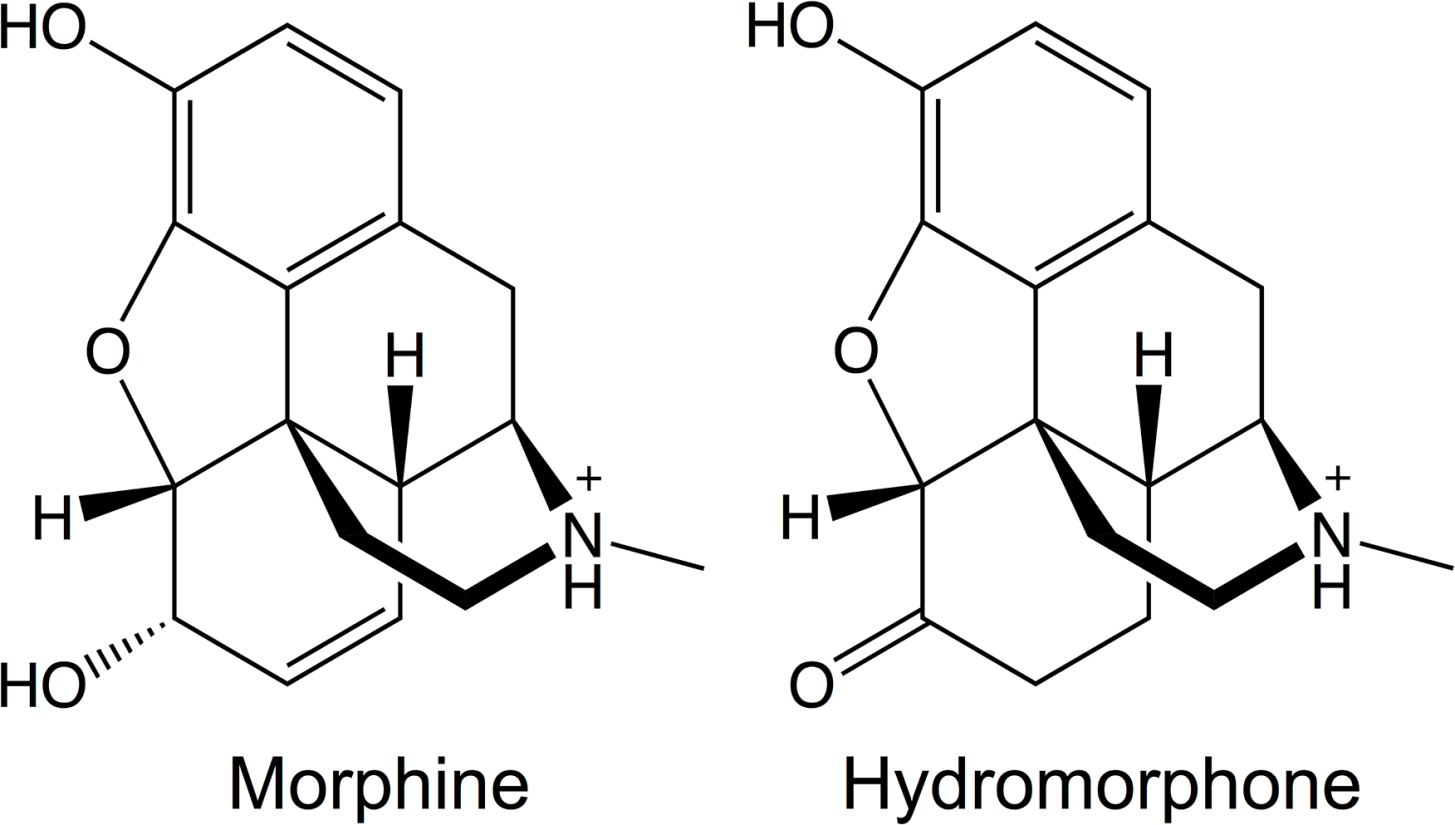
Molecular structures of morphine and hydromorphone.

Characterizing opioid-μOR interactions may help to understand how different compounds can trigger distinct downstream responses and cause the selective activation of particular signaling and regulatory pathways. This knowledge can be in turn exploited to develop novel potent analgesics lacking some of the undesirable properties of current opioids by activating specific μOR signaling pathways. Within this perspective, here we investigate the binding determinants and energetics of two prototypical opioid agonists, morphine (MOP) and hydromorphone (HMP), to μOR. The two agonists’ functional activities at the receptor differ. The EC_50_ values for cAMP (G-protein mediated responses) and for β-arrestin2 (Table 1) point to 3~4-fold increase in the potency of HMP relative to MOP [17]. By contrast, the agonists’ structural features (Fig 1) and binding affinities for μOR are very similar (Table 1). In this paper, we report structural features of the two agonists’ binding modes, along with the agonist-induced conformational changes in μOR. These changes might be present in the early steps toward an active state of the receptor.

**Table 1:** EC_50_ (nM) cAMP and β-arrestin2 values for MOP and HMP on human μOR [17], as well as the binding affinities of the two agonists for μOR.

	*K*_i_ (nM)	EC_50_ cAMP	EC_50_ β-arrestin2
MOP	8.0±3.6	50	501
HMP	4.0±0.8	16	126

The binding affinities are measured in this study (see “Materials and Methods” for details).

Our calculations are based on the X-ray structure of mouse μOR covalently bound with β-funaltrexamine (β-FNA), a semisynthetic opioid antagonist derived from morphine, resolved at 2.8 Å resolution (PDB entry: 4DKL) [18]. Mouse μOR shares 94% sequence identity with human μOR. Therefore, the 3D structure of mouse μOR provides an excellent model for investigating μOR-ligand interactions. We find that MOP and HMP bind to μOR with subtle differences that may account for their different binding affinities, which are consistent with *in vitro* experiments performed here.

## Materials and Methods

### Computational details

#### Structural models of the agonists in complex with μOR

We started from the X-ray structure of mouse μOR [18]. The structure contains a T4 lysozyme replacing the third intracellular loop (IL3), which is part of the putative G-protein binding epitope on class A GPCRs [19]. We constructed the missing IL3 using homology modeling by Modeller 9.9 [20]. In total 20,000 μOR models were generated, from which the one with the lowest Discrete Optimized Protein Energy (DOPE) score [20] was selected. The water molecules in the X-ray structure were preserved during the homology-modeling step. The NQ-Flipper [21] and H++ [22] webservers were used to examine the asparagine and glutamine side-chain rotamers and to predict the histidine side-chain protonation states, respectively. MOP and HMP were built and docked into the orthosteric binding site of μOR using MOE 2012.10 [23]. The protonation state of the side-chain residues forming μOR was estimated by the Protonate3D algorithm [24]. The tertiary amines of the ligands were protonated as they should be at physiological pH (Fig 1) and rotatable bonds were allowed to rotate. Docking was realized using an induced fit procedure that allows taking into account the flexibility of the protein side-chains located in the binding pocket. The electrostatic solvation energy was used to score the resulting binding modes using a generalized born/volume integral method [25]. From the top-ranked 25 modes of each ligand, we selected the mode in best agreement with available experimental data (see S1 File for details).

#### Embedding in membrane and solvation.

The DOWSER program [26] was used to detect and prefill possible hydrophilic cavities inside the MOP-μOR, HMP-μOR and β-FNA-μOR complexes. The complexes were oriented according to the OPM (Orientations of Proteins in Membranes) database [27] and then embedded in a bilayer of 1-Palmitoyl-2-oleoyl-sn-glycero-3-phosphocholine (POPC), the most abundant phospholipid in animal cell membranes [28]. We used the ‘Stockholm Lipids’ parameters that have been shown to be compatible with AMBER force fields [29], and the POPC bilayer configuration pre-equilibrated in water at 303 K [30]. The embedding was performed using the Inflategro2 program [31]. Each system was then immersed in a periodic 64×67×113 Å^3^ box, which ensured a distance always larger than 20 Å between adjacent images of the protein during the simulation. The systems were solvated in explicit water and neutralized with 0.15 M NaCl, resulting in ca. 8,430 water molecules, 23 Na^+^ and 37 Cl^-^ ions. Each system consisted of ca. 42,700 atoms in total.

#### Molecular Dynamics simulations

Energy minimizations and MD simulations were carried out using Gromacs 4.6 [32]. The TIP3P water model [33], the Åqvist parameters [34], and the Amber99SB_ILDN force field [35] were used for the water molecules, the ions, and the protein, respectively. As for the parameters of the ligands, we calculated their partial atomic charges at the DFT level with the HF/6-31G* basis set using Gaussian 09 [36]. The obtained electrostatic potential was fitted by the RESP program [37] in Amber11 to generate atomic point charges (provided in S2 File). The other parameters were taken from the General Amber force field (GAFF) [38] to build the topology of the ligands (S2 File). The ligand-μOR complexes underwent 1,000 steps of steepest-descent energy minimization with 5,000 kcal·mol^-1^·Å^-2^ harmonic positional restraints on the protein-ligand complex, followed by 2,500 steps of steepest-descent and 2,500 steps of conjugate-gradient minimization without restraints. Then, the systems were gradually heated to 300 K in 6 steps of 100-ps MD simulations (from 2 K to 60 K, 120 K, 180K, 240 K and 300 K). The velocities were generated consistent with a Maxwell-Boltzmann distribution at the corresponding temperature. Each system underwent 5 ns equilibration and 0.8-μs production MD simulations in the NPT ensemble (*P* = 1 bar, *T* = 300 K) by using the Andersen-Parrinello-Rahman barostat [39, 40] and the Nose-Hoover thermostat [41]. Semi-isotropic pressure coupling was applied to allow the simulation box in the *z*-axis (perpendicular to the lipid bilayer) to vary independently of the *x-y* plane. The LINCS algorithm [42] was employed to constrain all the bond lengths. Van der Waals and short-range electrostatic interactions were cut off at 12 Å. Long-range electrostatic interactions were computed using the Particle Mesh Ewald summation (PME) [43] method with a Fourier grid spacing of 1.2 Å. A 2-fs time step was used in the MD calculations. Using the same procedure, 0.5-μs additional MD simulations for each system were obtained starting from different initial velocities.

#### Free energy calculations

**Fig 2:**
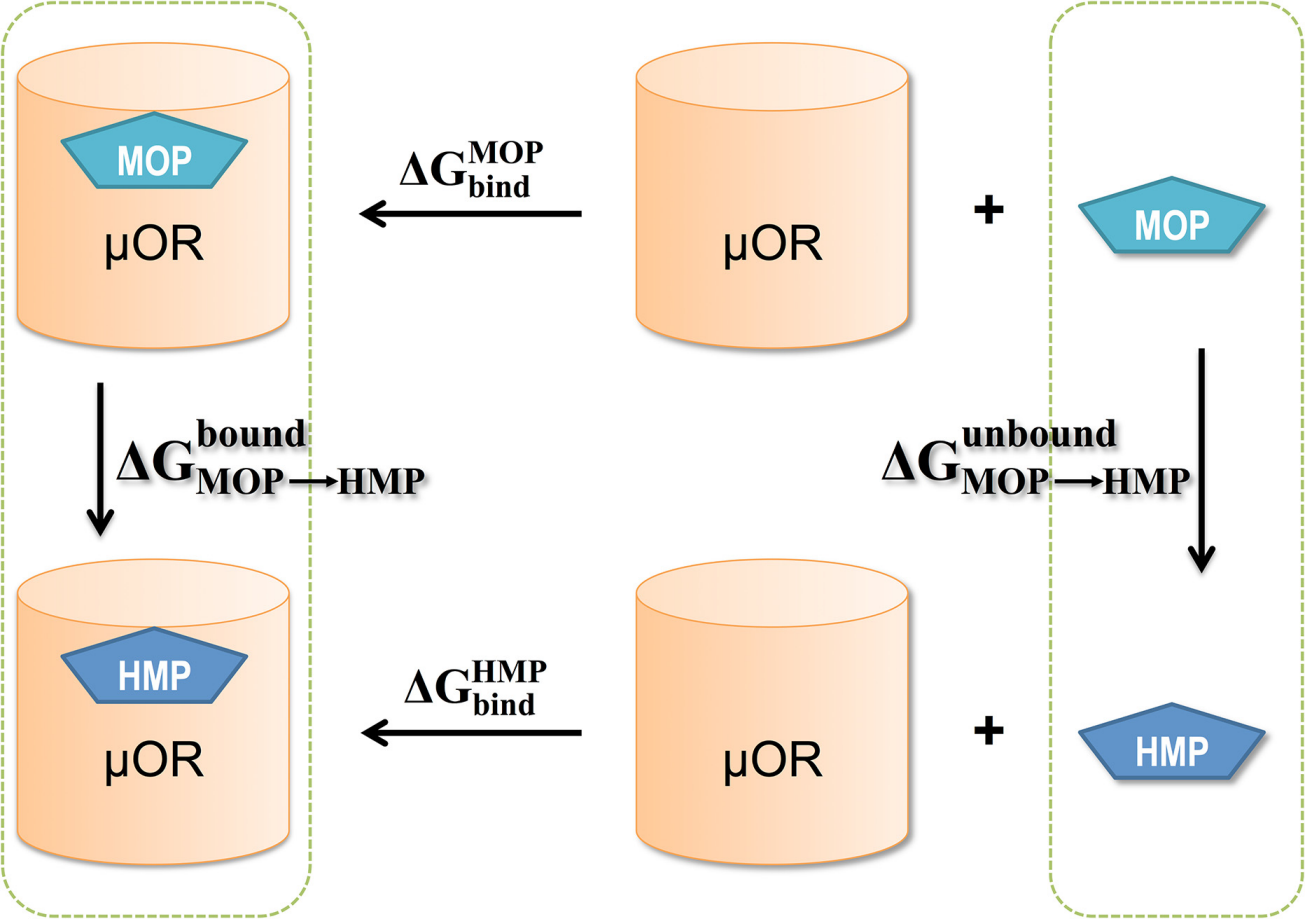
The thermodynamic cycle for computing the free energy difference between MOP and HMP upon binding to μOR: $\Delta \Delta G_{\text{bind}}=\Delta G_{\text{bind}}^{\text{MOP}}-\Delta G_{\text{bind}}^{\text{HMP}}=\Delta G_{\text{MOP}\to \text{HMP}}^{\text{bound}}-\Delta G_{\text{MOP}\to \text{HMP}}^{\text{unbound}}.$ The unbound state requires transformation of the ligands alone in solution, since the receptor is the same in both cases.

Alchemical free energy perturbation (FEP) methodologies were employed to estimate the difference in the binding free energy to μOR, *∆∆G*_bind_, between MOP and HMP. Using the thermodynamic cycle in Fig 2, *∆∆G*_bind_ from MOP to HMP can be calculated as the free energy difference between two alchemical pathways: the transformation of MOP to HMP while bound to μOR, and that of MOP to HMP in solution (unbound). We performed alchemical transformation from MOP to HMP and *vice versa*.

The transformations in solution (the unbound state) started from the agonists pre-equilibrated in a periodic 50×50×50 Å^3^ box containing ca. 4,120 water molecules and a Cl^-^ counterion. The force field parameters and the simulation procedure were the same as those used above. For each agonist, 5 ns pre-equilibration MD was carried out. The transformations of the agonists bound to μOR (the bound state) were started from representative configurations obtained from a cluster analysis of the agonist-μOR trajectories using g_cluster in Gromacs tools [44] and the Gromos method [45].

The transformations from one agonist (A) to the other (B) were performed in the following steps: (i) charge annihilation by scaling the electrostatic potential energy of A, in 30 steps (*∆λ* = 0.025 for the *λ* values from 0 to 0.25 and from 0.8 to 1, and *∆λ* = 0.05 for the *λ* values from 0.25 to 0.8); (ii) decoupling the van der Waals interactions and transforming A into B in 25 steps (*∆λ* = 0.01 for the *λ* values from 0 to 0.05, and *∆λ* = 0.05 for the *λ* values from 0.05 to 1); and (iii) charge generation for B by scaling its electrostatic potential energy in 30 steps (using the same *λ* values as those in (i)). The simulations in the unbound state at each *λ* value included 5,000 steps of steepest descent energy minimization and 5,000 steps L-BFGS energy minimization, 200 ps NVT (*T* = 300 K) equilibration, 1 ns NPT (*P* = 1 bar, *T* = 300 K) equilibration and 10 ns NPT (*P* = 1 bar, *T* = 300 K) production run with dH/d*λ* collection every 10 steps. The temperature was controlled via Langevin dynamics and the pressure, Andersen-Parrinello-Rahman barostat [39, 40]. The simulations in the bound state were performed in the same manner except that the timescales in this case were: 500 ps NVT equilibration, 5 ns NPT equilibration and 10 ns NPT production run.

The free energies of the steps involved in the transformations were calculated using the Bennett’s acceptance ratio method (BAR) [46] implemented in Gromacs 4.6. BAR combines the information normally used for forward and reverse free energy perturbations. The free energy difference of a transformation is a sum over those of *n* successive *λ* values, $\Delta G=\sum_{i=0}^{n-1}\delta G_{i}$, in which *δG*_*i*_ is computed iteratively according to

$${\langle {\left[1+e^{-\beta \left(U_{\lambda_{i}+1}\left(x_{i}\right)-U_{\lambda_{i}}\left(x_{i}\right)-\delta G_{i}\right)}\right]}^{-1}\rangle }_{\lambda_{i}}={\langle {\left[1+e^{-\beta \left(U_{\lambda_{i}+1}\left(x_{i+1}\right)-U_{\lambda_{i}}\left(x_{i+1}\right)+\delta G_{i}\right)}\right]}^{-1}\rangle }_{\lambda_{i}+1},$$

where *U* is the potential energy as a function of the system’s configuration, *x*.

### Experimental details

*Human μOR binding assay*. The human μOR binding assay was performed as homogeneous SPA-assay (scintillation proximity assay) using the assay buffer 50 mM TRIS-HCl (pH 7.4) supplemented with 0.052 mg/ml bovine serum albumin (Sigma-Aldrich Co., St. Louis, MO). The final assay volume (250 μl/well) included 1 nM of [N-allyl-2,3-^3^H]naloxone as ligand (PerkinElmer Life Sciences, Inc. Boston, MA, USA), and either test compound in dilution series or 25 μM unlabeled naloxone for determination of unspecific binding. The test compound was diluted with 25% DMSO in H_2_O to yield a final 0.5% DMSO concentration, which also served as a respective vehicle control. The assay was started by adding wheat germ agglutinin coated SPA beads (GE Healthcare UK Ltd., Buckinghamshire, UK), which had been preloaded with human μOR membranes (PerkinElmer Life Sciences, Inc. Boston, MA, USA). After incubation for 90 minutes at RT and centrifugation for 20 minutes at 500 rpm the signal rate was measured by means of a 1450 Microbeta Trilux β-counter (PerkinElmer Life Sciences/Wallac, Turku, Finland). Half-maximal inhibitory concentration (IC50) values reflecting 50% displacement of [^3^H]naloxone-specific receptor binding were calculated by nonlinear regression analysis and Ki values were calculated by using the Cheng-Prusoff equation [47].

## Results and Discussion

The initial binding modes of MOP and HMP obtained from docking are very similar (see S1 File for details). During 0.8 μs MD simulations, the transmembrane domain of μOR remain relatively rigid (Cα RMSF ranging from 0.4 Å to 1.5 Å) while, as expected, the loop regions of μOR display larger fluctuations (Cα RMSF ranging from 1.0 to 7.1 Å) (S1 Fig). The largest fluctuations occur in the modeled IL3, the region where a T4-lysozyme is located in the X-ray structure of the receptor, for the case of the HMP-μOR complex (S1 Fig). The Cα RMSD of μOR transmembrane domain are 1.7±0.3 Å, 1.3±0.2 Å and 1.2±0.1 Å in the MOP-μOR, HMP-μOR and β-FNA-μOR complexes, respectively, well below the resolution of the X-ray structure (S2 Fig). MOP and HMP mostly maintain their initial docking mode, while β-FNA well preserves its binding mode in the X-ray structure, the salt-bridge with D147 being preserved during the timescale of all the MD simulations. The RMSD of the ligand non-hydrogen atoms are 3.3±0.4 Å, 2.0±0.4 Å and 1.6±0.2 Å for MOP, HMP and β-FNA, respectively (S2 Fig).

**Fig 3:**
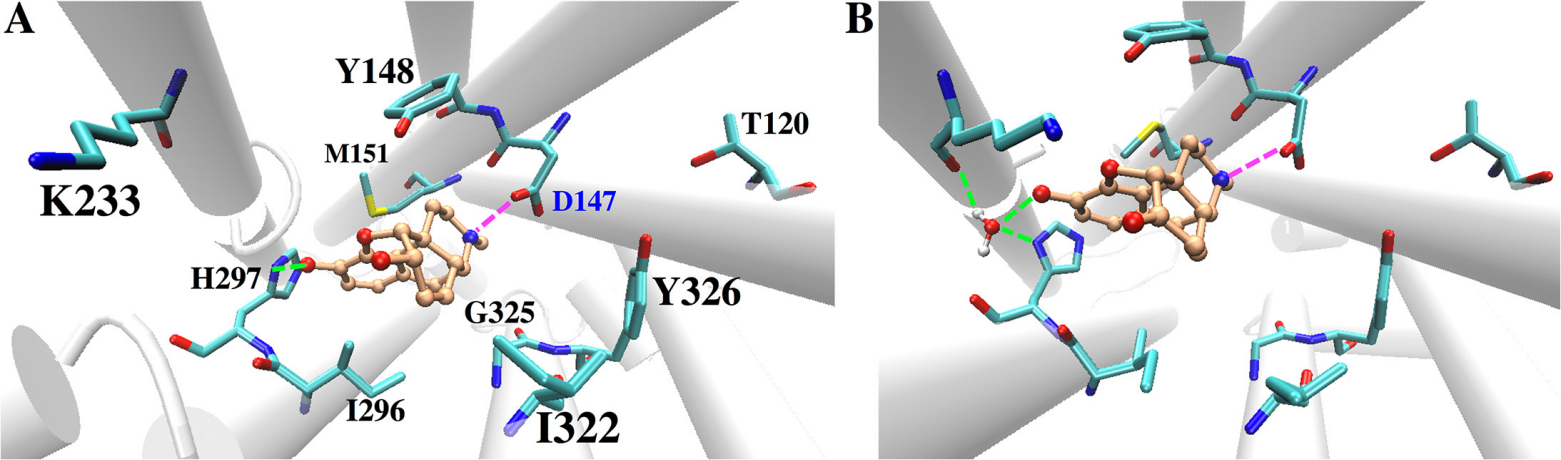
Representative structure of the MD simulations for (A) the MOP-μOR and (B) the HMP-μOR complexes obtained from clustering analysis. The ligand carbon atoms are in orange. H-bonds and salt-bridges are shown in green and magenta dashed lines, respectively. For clarity hydrogen atoms of the ligands and the μOR residues are not shown. H297 is monoprotonated at the Nε atom.

**Fig 4:**
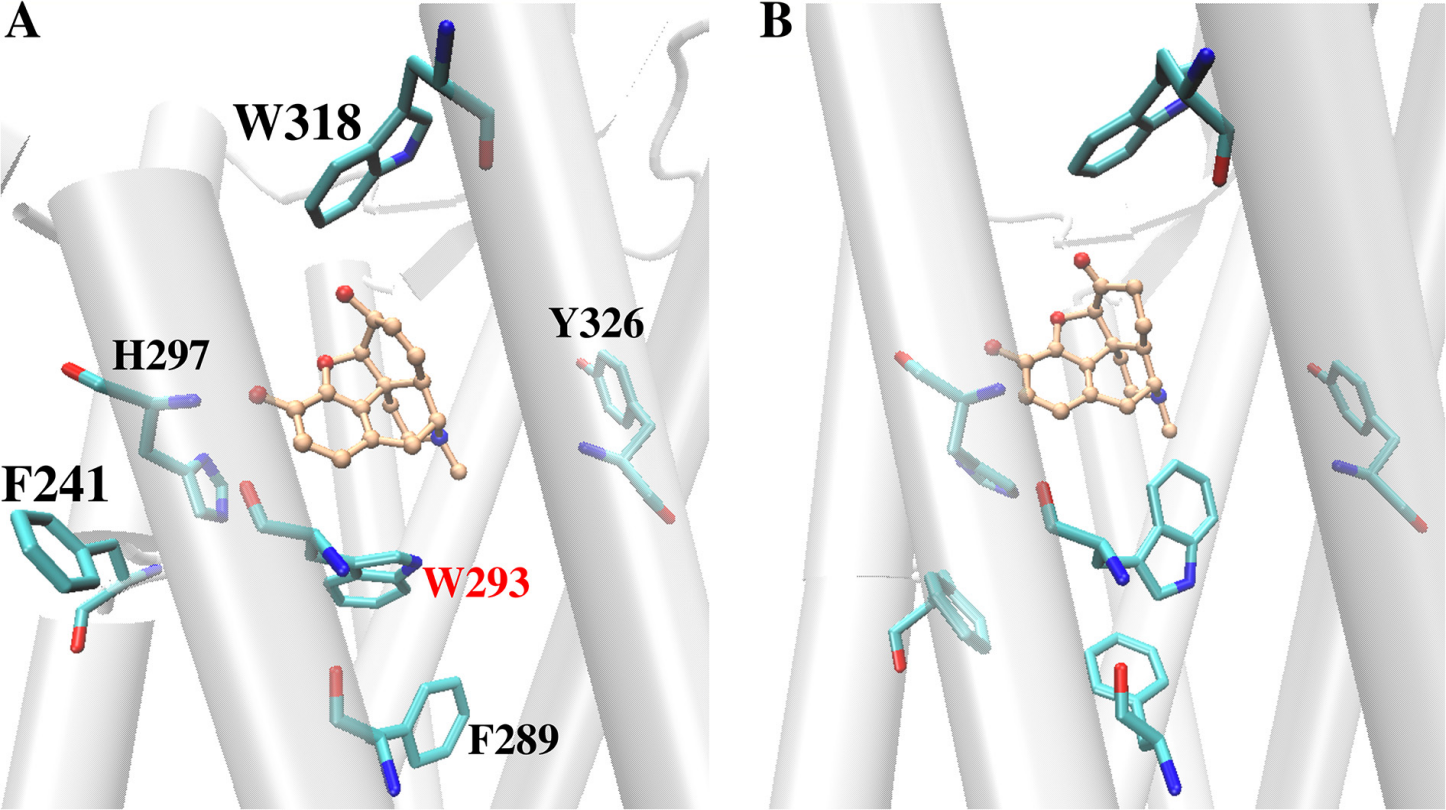
Arrangements of aromatic residues at the μOR orthosteric binding site upon binding with (A) MOP and with (B) HMP.

The two agonists (MOP and HMP) accommodate into the active site cavity in a slightly different manner, forming different H-bond patterns with residue H297 in TM6. In particular, MOP establishes a direct H-bond with H297 side chain, whereas HMP maintains water-mediated H-bonds with H297 side chain and K233 backbone mostly via one bridging water molecule (Fig 3 and S3 Fig). The presence of a cyclohexanone moiety within the HMP skeleton as opposed to a cyclohexenol ring, which is its cyclic counterpart in MOP, determines this different interaction pattern as it alters the drug surface available for hydrophobic interactions with protein residues in this region and slightly modifies the conformation of the polycyclic structure. MOP fits into the active site cavity establishing additional van der Waals contacts with G325, I296 and M151 while disrupting the initial interaction between D147 and the Y326 hydroxyl group. This disruption occurs at ~220 ns when Y326 moves away from D147 and eventually forms a very stable H-bond with T120. On the contrary, HMP binding causes the flip of the W293 side chain during the first 225 ns and gives rise to a stable interaction between the W293 and Y326 side chains (Fig 4). As a consequence, Y326 remains in close proximity to the ligand and preserves a H-bond with D147 (Fig 4B). The flip of the W293 side chain also leads to a rearrangement of the nearby aromatic residues, which establish different van der Waals contacts than those in MOP-μOR (Fig 4). The W293 reorientation separates HMP from G325 and places the ligand closer to I322, resulting in slightly different HMP-μOR hydrophobic contacts from those between MOP and the receptor (see Table 2). In addition, the flip of the W293 side chain allows more water molecules to enter the binding cavity below HMP (i.e. opposite to the extracellular side). After W293 flips, 15±2 water molecules are present inside the cavity of HMP-µOR, to be compared with 10±2 in MOP-μOR (S3 File). The different degrees of hydration are statistically significant, as indicated by normalized histograms and Welch’s *t*-test on the data (S3 File). Additional 0.5-μs MD simulations of MOP-μOR and HMP-μOR, starting from different initial velocities, reproduce the above-observed differences of the H-bond pattern, the W293 side chain orientation, the van der Waals contacts and the hydration in the binding cavity (S3 File).

**Table 2:** Minimum distances between both morphinan drugs (MOP and HMP) and selected protein residues.

	I296	M151	G325	I322
MOP	2.4 ± 0.2	2.2 ± 0.2	2.4 ± 0.3	3.3 ± 0.5
HMP	2.4 ± 0.2	2.3 ± 0.2	5.0 ± 0.3	2.7 ± 0.2

Distances are given in Å and averaged over the finite temperature MD trajectories.

**Fig 5:**
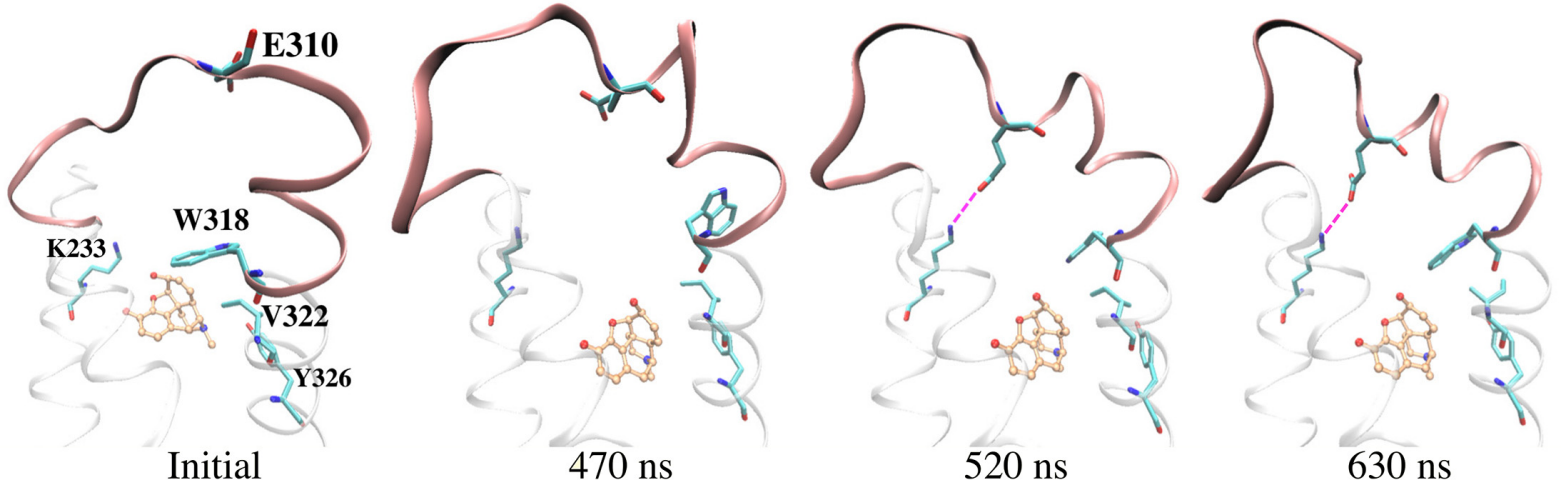
Conformational change of μOR EL3 observed in the case of MOP binding. E310 in EL3 forms a salt-bridge with K233 (magenta dashed lines), which remains until the end of the simulation.

In addition, our MD simulations show the impact of agonist binding on the flexibility of the extracellular loop 3 (EL3). MOP binding is associated with conformational changes in this loop (Fig 5). Notably, the presence of MOP in the active site pocket causes W318 side chain to move away from the ligand after 470 ns, which allows E310 in EL3 to form salt-bridge interactions with K233 that reduces EL3 mobility (Fig 5).

Therefore, our MD simulations confirm the key role of D147 and H297 in binding opioid ligands [48–50] and illustrate subtle differences in the binding mode of MOP and HMP, which may account for the slightly difference in their binding affinity. Indeed, HMP accommodates in the binding pocket slightly differently from MOP, maintaining a larger propensity of water-mediated H-bonds with H297 and K233 rather than a direct H-bond with H297. HMP also leads to slightly higher hydration in the binding pocket than MOP.

Next, we calculate the relative binding affinities of MOP and HMP to μOR using alchemical free energy perturbation. This approach is highly suitable for these complexes as the ligands involved in the alchemical transformation are structurally and chemically similar (Fig 1) and, therefore, one ligand can smoothly and gradually be transformed into the other. Moreover, the salt-bridge between the positively charged basic nitrogen atom of the morphinan drugs and the negatively charged D147 is preserved during our simulations. It acts as an anchor, which limits ligand diffusion and allows adequately sampling of all the relevant drug conformations inside the active site cavity. This fact significantly reduces the statistical error associated to this kind of calculations. We critically evaluate the convergence of the computed transformation by examining the hysteresis resulting from the FEP calculations of both the forward (MOP to HMP) and the reverse (HMP to MOP) transformations. This turns out to be very satisfactory (Table 3). Notably, the accommodation of both agonists inside the μOR active site is fully consistent with that found during the standard MD simulations of the MOP-μOR and HMP-μOR complexes. In particular, the H-bond pattern emerged from both forward and reverse alchemical transformations is consistent with that in the standard MD simulations, i.e. water-mediated H-bonds between HMP and H297, and direct H-bond between MOP and H297 (S3 Fig). As in the MD, the W293 side chain orientation differs in the complexes with HMP and with MOP during the alchemical free energy perturbation (S3 File). Therefore, the forward and backward alchemical transformations further corroborate the findings emerging from the MD that the differences in the H-bond patterns, the van der Waals contacts and the W293 side chain orientations are significant and are not statistical fluctuations. Instead, the aforementioned EL3 conformational changes observed in the MD simulations of MOP-μOR are not present in the alchemical transformations. Thus, this feature is possibly due to statistical fluctuations and may contribute insignificantly to the ligand binding affinity.

**Table 3:** Free energy differences (in kcal/mol) for the described transformations.

	Bound state	Unbound state (in solution)	*∆∆G*_bind_	Exp. ^a^
MOP→HMP	16.7 ± 0.6	-17.9 ± 0.5	-1.2 ± 1.1	-0.4±0.3
HMP→MOP	-17.5 ± 0.6	18.3 ± 0.2	0.8 ± 0.8	0.4±0.3

Error estimates are included.

^a^ Calculated as $\Delta \Delta G_{\text{MOP}\to \text{HMP}}^{\text{exp}.}=\Delta G_{\text{bind}}^{\text{HMP}(\text{exp}.)}-\Delta G_{\text{bind}}^{\text{MOP}(\text{exp}.)}=RT\;\text{ln}\left[K_{i}^{\text{HMP}(\text{exp}.)}/K_{i}^{\text{MOP}(\text{exp}.)}\right].$

Despite the relatively large standard deviations of our free energy calculations, the calculated and measured differences in binding affinity are in qualitative agreement. However, because these differences are very small and are close to being non-significant, these results must be interpreted with caution. Exhaustive computational studies on multiple ligands, such as those in refs. [51, 52] for the glutamate receptor, would be required to establish quantitative conclusions on ligand-μOR energetics.

**Fig 6:**
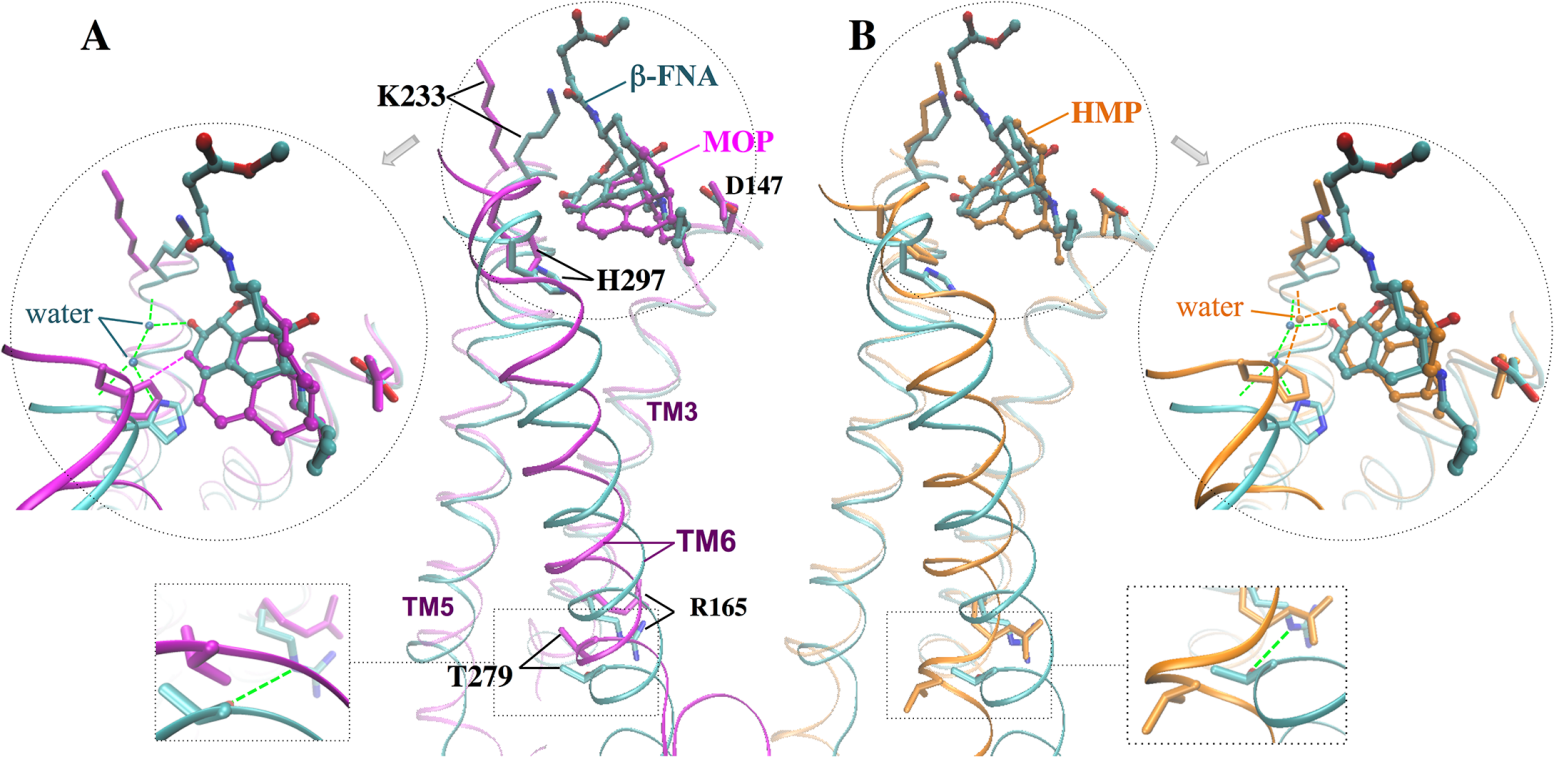
Superimposition of representative MD structures obtained by cluster analysis (see Materials and Methods) for (A) MOP-μOR and (B) HMP-μOR onto the X-ray structure of β-FNA-μOR [18]. Dashed lines indicate H-bonds.

Finally, we compare the two agonists with β-FNA. The three ligands feature very similar polycyclic ring skeletons and a common salt bridge with D147. However, β-FNA shows different H-bond pattern: two water molecules mediate the ligand’s H-bonds with H297 and K233, which are preserved throughout the two independent MD simulations. Such pattern is not retained in either of the two agonists (Fig 6). This is likely due to the covalent bond between β-FNA and K233, which constraints the antagonist’s location and mobility in the binding pocket.

We close this section by comparing the available experimental structural information on the antagonist-bound receptor with the structural predictions emerging from our simulations. In the X-ray structure of the antagonist β-FNA covalently bound to μOR [18]–so far the only structure for this receptor–R165 in TM3 forms a H-bond with T279 in TM6 (Fig 6) [18], which turns out to be very stable during the MD simulation (lifetime 89%). By contrast, the presence of the two agonists in our simulations cause a loss of this key H-bond (lifetime < 2% in both cases) along with a translational shift of TM6 with respect to TM3 (Fig 6). TM6 moves toward TM5 and, more, to the extracellular side. The Cα RMSDs of TM6 with respect to the X-ray structure are 3.0±0.5 Å, 2.3±0.5 Å and 1.2±0.3 Å for MOP-μOR, HMP-μOR and β-FNA-μOR, respectively; whereas those of TM3 are 0.6±0.1 Å, 0.6±0.1 Å and 0.5±0.1 Å, respectively (S4 Fig). Hence, we suggest that this shift, along with the loss of the R165-T279 H-bond, is a specific feature of an active-like state. Our hypothesis is fully consistent with the following facts: (i) the T279K mutation gave rise to a constitutively active µOR variant [53]; (ii) R165 is conserved in 98% of class A GPCRs and is part of the D(E)RY motif found at the intracellular end of TM3, conserved across ~70% of class A GPCRs [54]; (iii) the R165-T279 H-bond may stabilize the receptor in an inactive state [18, 53], similar to the role of the so-called ‘ionic lock’–a salt bridge in the equivalent position between TM3 and TM6 in rhodopsin [55]; (iv) previous structural studies have suggested an activation mechanism across GPCRs based on a global toggle switch mechanism involving similar movements of TM6: this helix would move toward TM5 while increasing its separation from TM3 [56]. A common activation mechanism has been proposed for class A GPCRs based on a number of X-ray structures of active and inactive GPCRs [57]. The mechanism involves a significant outward movement of the intracellular end of TM6. Hence, the relatively small movement between this helix and TM3 observed here might be an early event toward the activation.

**Fig 7:**
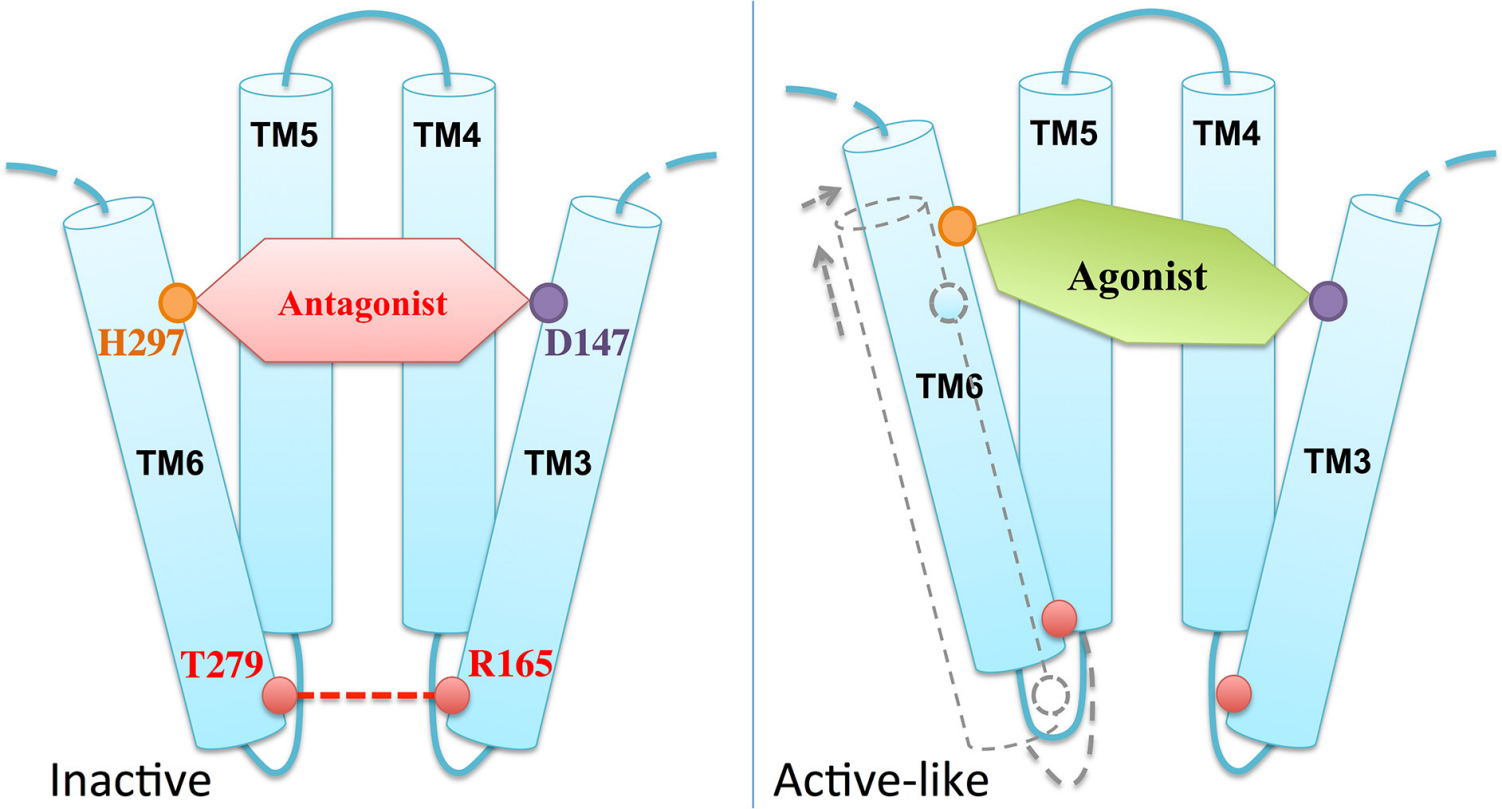
Schematic model of the agonist-induced μOR conformational change into an active-like state.

Comparison of the structural determinants at the active site suggests that H297 at TM6 plays an important role in the agonist-induced shift of TM6. H297 forms direct or water-mediated H-bonds as well as van der Waals interactions with the two agonists, consistent with its role as an “anchor” for opioids binding to μOR [50]. However, the agonists stabilize the residue in a position that is rather shifted relative to that in the β-FNA-μOR complex (Fig 6). Hence, we suggest that the ligand-H297 interaction is a key factor for the displacement of TM6 relative to TM3 (Fig 7).

## Conclusions

In the present work, we investigated the binding characteristics of two morphinan agonists (MOP and HMP) to μOR by molecular simulations. Comparison is made with a simulation of the antagonist β-FNA covalently bound to μOR. An atomistic description of the ligand-receptor interactions was obtained using 3.9-μs MD simulations and 1.7 μs alchemical free energy perturbation calculations. The MOP binding mode obtained here is consistent with previous 5.6 μs long MD simulations [58]. Our simulations point to the crucial role of D147 and H297, which anchor the agonists via highly stable salt-bridge and H-bond interactions, respectively. MOP forms a direct H-bond with H297 whereas HMP forms water-mediated H-bonds with H297, both of which differ from the H-bond pattern of β-FNA. HMP also forms water-mediated H-bonds with K233. The two agonists form slightly different van der Waals contacts with residues W293, I322 and G325. W293 side chain adopts distinct orientations upon MOP and HMP binding. The HMP-bound pocket is slightly more hydrated than in the MOP-bound one. These subtle differences in the agonists’ binding mode likely account for the small difference in their binding affinity, which is here measured *in vitro* and calculated with alchemical free energy perturbation. Both agonists alter the relative position between TM3 and TM6 of the receptor, likely via their interactions with H297 in TM6. The alteration disrupts the R165-T279 H-bond between TM3 and TM6 that has been suggested to stabilize the receptor in an inactive conformation in the X-ray structure [18]. By contrast, in the simulation of β-FNA-μOR the relative position between TM3 and TM6 as well as the R165-T279 H-bond remains similar to those in the X-ray structure [18].

In conclusion, this study characterizes the structural features accounting for the two agonists’ different affinities, and more importantly identifies the key agonist-receptor interactions that likely promote receptor activation.

## Supporting Information

S1 Fig RMSF of μOR Cα atoms during the MD simulations of MOP-μOR (blue) and HMP-μOR (orange). (TIFF)

S2 Fig RMSD of μOR Cα atoms (blue) and of the ligand non-hydrogen atoms (red) with respect to the initial structure during the 0.8 μs MD simulations of (left) MOP-μOR, (middle) HMP-μOR and (right) β-FNA-μOR. (TIFF)

S3 Fig H-bond patterns between the agonists and μOR residue H297: direct (blue) and water-mediated (red) H-bonds. **(A)** The dominant H-bond patterns during the MD simulations for MOP-μOR and HMP-μOR. The number of bridging water molecules in these H-bonds is plotted in **(B, C)** as a function of simulation time in the two independent MD simulations (lasting 0.8 μs and 0.5 μs, respectively), and in **(D)** during the course of the forward and backward alchemical transformations.
(TIFF)

S4 Fig RMSD of μOR TM6 Cα atoms in the case of MOP bound (blue), HMP bound (orange) and β-FNA bound (green) with respect to those in the X-ray structure. (TIFF)

S1 File MOP and HMP binding modes selected from docking. (PDF)

S2 File Partial charges and atom types of the ligands used in the MD simulations. (PDF)

S3 File Differences between MOP-μOR and HMP-μOR in the W293 side chain orientation, the hydration inside the binding cavity and the van der Waals contacts. (PDF)
