# Supplementary figures and images for: Structural Determinants for the Binding of Morphinan Agonists to the μ-Opioid Receptor
Source: PLoS One. 2015 Aug 17;10(8):e0135998. doi: 10.1371/journal.pone.0135998 (PMC4539194; doi:10.1371/journal.pone.0135998)

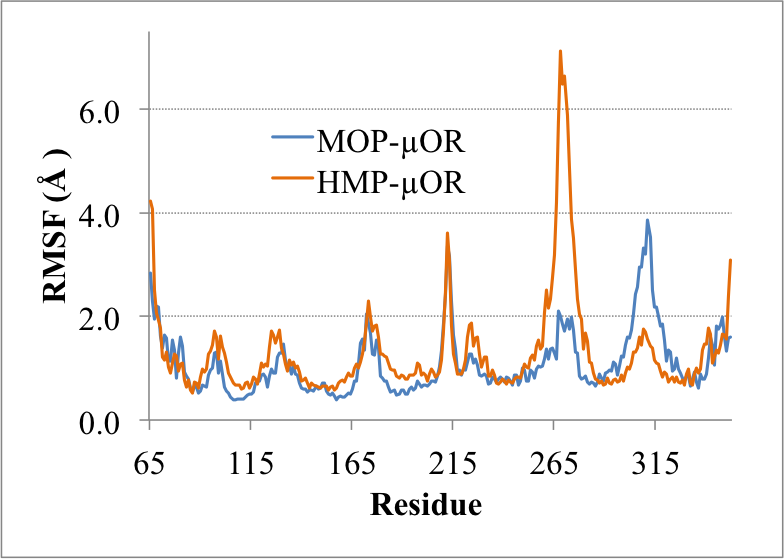

Supplement: S1 Fig — (TIFF) [file pone.0135998.s001.tiff]

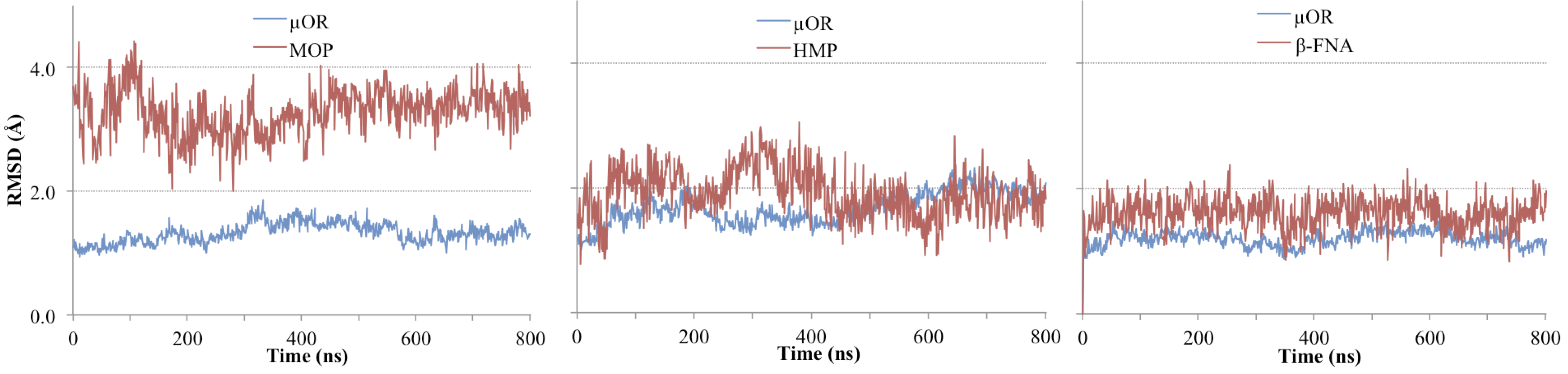

Supplement: S2 Fig — (TIFF) [file pone.0135998.s002.tiff]

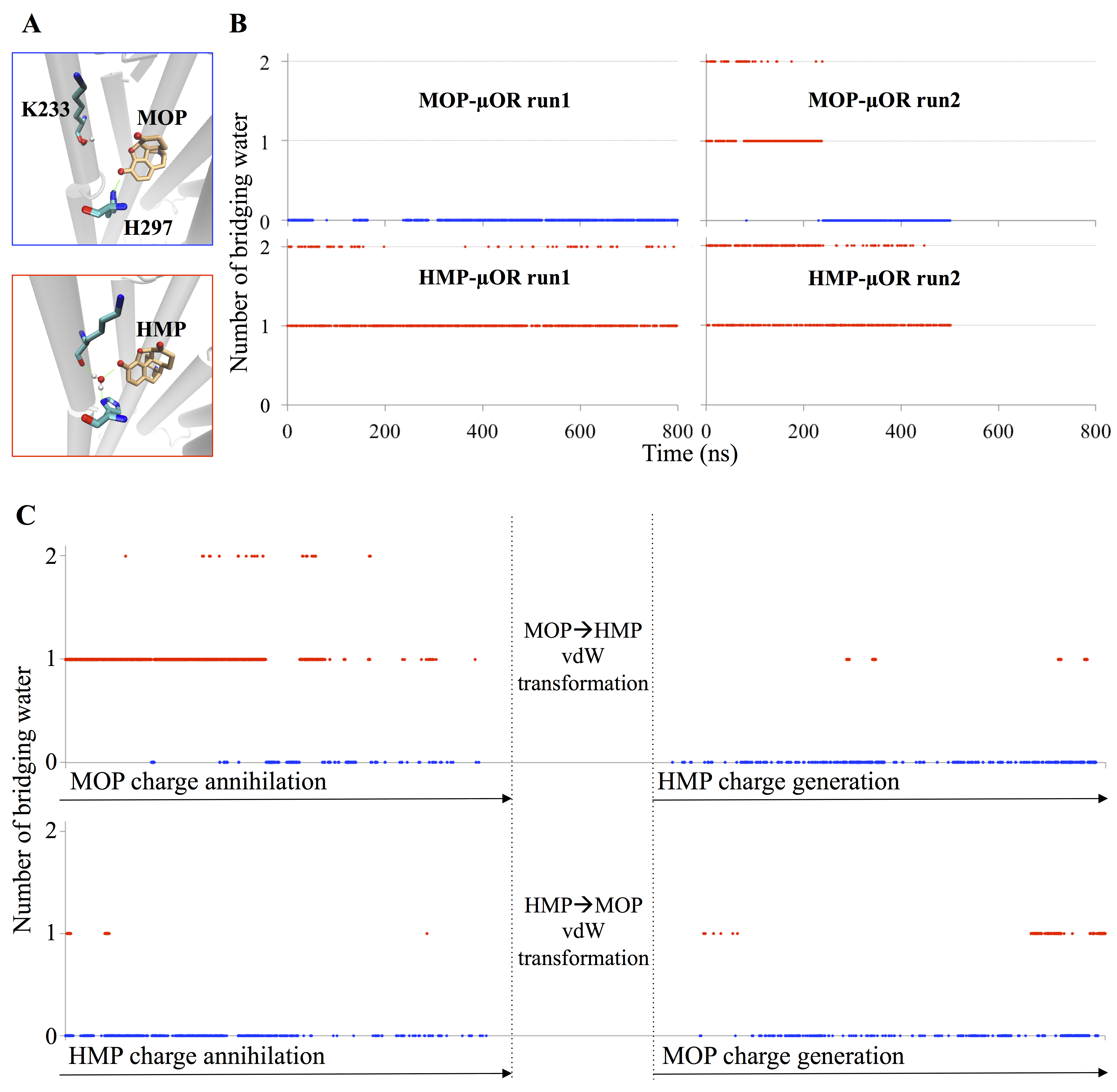

Supplement: S3 Fig — (A) The dominant H-bond patterns during the MD simulations for MOP-μOR and HMP-μOR. The number of bridging water molecules in these H-bonds is plotted in (B, C) as a function of simulation time in the two independent MD simulations (lasting 0.8 μs and 0.5 μs, respectively), and in (D) during the course of the forward and backward alchemical transformations. (TIFF) [file pone.0135998.s003.tiff]

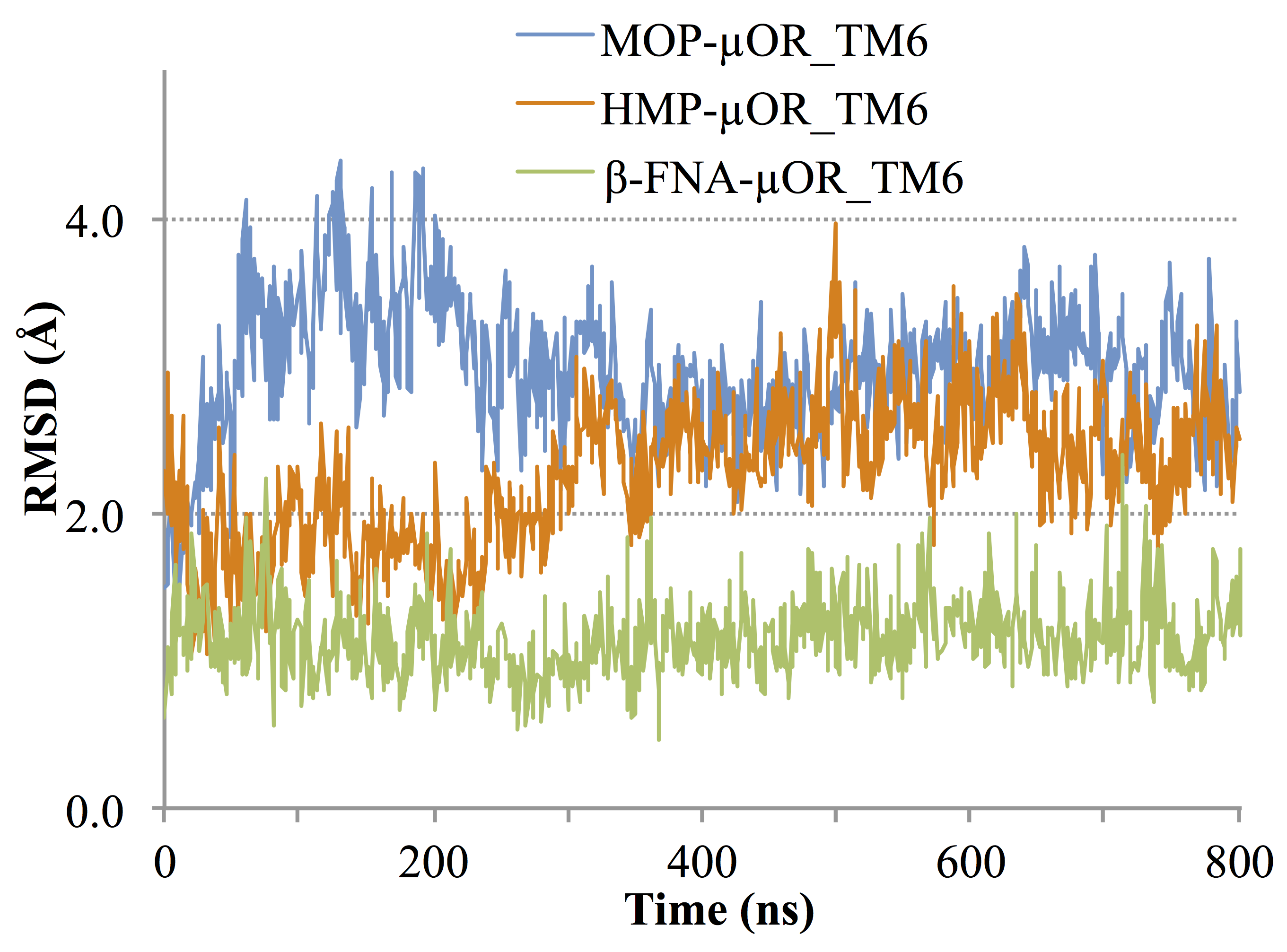

Supplement: S4 Fig — (TIFF) [file pone.0135998.s004.tiff]
