# Supplementary material for: Structural Determinants for the Binding of Morphinan Agonists to the μ-Opioid Receptor
Source: PLoS One. 2015 Aug 17;10(8):e0135998. doi: 10.1371/journal.pone.0135998 (PMC4539194; doi:10.1371/journal.pone.0135998)
Supplement: S1 File — (PDF) [file pone.0135998.s005.pdf]

MOP and HMP binding modes selected from docking. We selected the ligands' binding modes that on the one hand resemble as close as possible the mode of the structurally similar β-FNA in the X-ray structure [1]. On the other hand, the selected modes form direct contacts with most (8 out of 9) residues that play important roles in MOP binding, as identified by molecular biology experiments (Table S1, Fig. S1). In particular, both agonists form a salt-bridge with D147 and water-bridged H-bond interactions with H297, consistently with the fact that residues D147 and H197 have been found to be crucial for 'anchoring' opioids in the μOR binding pocket [2, 3]. The only residue not in contact with the ligands is H319, which is located relatively far from the binding pocket. The fact that H319A mutation reduces drastically MOP binding [4] likely indicates indirect contribution of H319 to binding the agonist. In the X-ray structure, similarly, H319 side chain points outward the binding pocket without direct interaction with β-FNA [1]. Finally, our selected binding modes resemble closely to that found in previous μs-scale MD simulations of the MOP-μOR complex [5].

Table S1: Key residues for binding MOP identified by site-directed mutagenesis in human, mouse or rat μOR [a].

	**Wild-type**	**Mutants**			
Ref. [6]	19.6±3.0	W318L<br>89.0±19.0	W318K<br>187.0±85.0		
Ref. [2]	20.0±3.5	Y326F<br>118.9±23.0	H297A<br>Nearly no binding		
Ref. [7]	0.81±0.07	M151S<br>63.1±16.3	W293H<br>15.1±2.5	D147A<br>4.7±0.8	I296A<br>4.1±0.7
Ref. [4]	9.6±3.2	Y148F<br>29.3±9.0	H319A<br>100.0±10.0		

The binding affinities are given in nanomolar together with standard errors.

[a] Mouse and rat μOR are very similar. They share 94% and 98% sequence identity with human μOR, respectively (sequence information from www.uniprot.org).

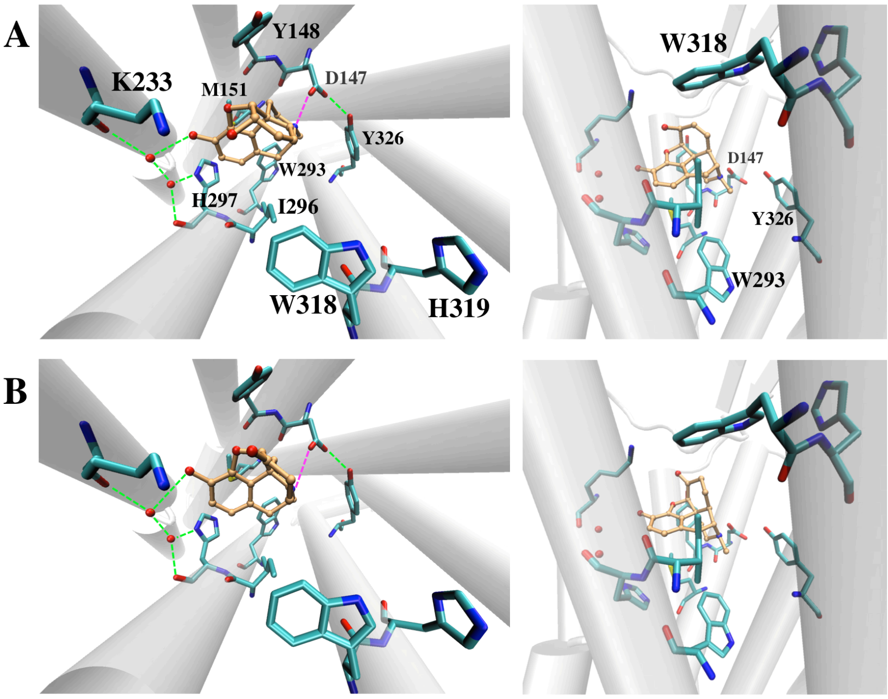

Fig. S1. The best binding mode of (A) MOP and (B) HMP in the orthosteric site of μOR from the extracellular view (left) and the side view (right). The receptor is shown in gray cartoon. The ligands and key binding-site residues are shown in ball-and-stick and licorice, respectively. Green and magenta dashed lines indicate H-bonds and salt-bridges, respectively. H297 is monoprotonated at the Nε atom.

Supporting References

1. Manglik A, Kruse AC, Kobilka TS, Thian FS, Mathiesen JM, Sunahara RK, et al. Crystal structure of the mu-opioid receptor bound to a morphinan antagonist. Nature. 2012;485(7398):321-6. Epub 2012/03/23. doi: 10.1038/nature10954. PubMed PMID: 22437502; PubMed Central PMCID: PMC3523197.
2. Mansour A, Taylor LP, Fine JL, Thompson RC, Hoversten MT, Mosberg HI, et al. Key residues defining the mu-opioid receptor binding pocket: A site-directed mutagenesis study. J Neurochem. 1997;68(1):344-53. PubMed PMID: ISI:A1997VY89300043.
3. Surratt CK, Johnson PS, Moriwaki A, Seidleck BK, Blaschak CJ, Wang JB, et al. -Mu Opiate Receptor - Charged Transmembrane Domain Amino-Acids Are Critical for Agonist Recognition and Intrinsic Activity. Journal of Biological Chemistry. 1994;269(32):20548-53. PubMed PMID: ISI:A1994PB31700054.
4. Xu H, Lu YF, Partilla JS, Zheng QX, Wang JB, Brine GA, et al. Opioid peptide receptor studies, 11: Involvement of Tyr148, Trp318 and His319 of the rat mu-opioid receptor in binding of mu-selective ligands. Synapse. 1999;32(1):23-8. doi: Doi 10.1002/(Sici)1098-2396(199904)32:1<23::Aid-Syn3>3.0.Co;2-N. PubMed PMID: ISI:000078855700003.
5. Yuan SG, Vogel H, Filipek S. The Role of Water and Sodium Ions in the Activation of the mu-Opioid Receptor. Angew Chem Int Edit. 2013;52(38):10112-5. doi: Doi 10.1002/Anie.201302244. PubMed PMID: ISI:000324309900052.

6. Bonner G, Meng F, Akil H. Selectivity of mu-opioid receptor determined by interfacial residues near third extracellular loop. European journal of pharmacology. 2000;403(1-2):37-44. Epub 2000/09/02. PubMed PMID: 10969141.
7. Serohijos AWR, Yin SY, Ding F, Gauthier J, Gibson DG, Maixner W, et al. Structural Basis for mu-Opioid Receptor Binding and Activation. Structure. 2011;19(11):1683-90. doi: Doi 10.1016/J.Str.2011.08.003. PubMed PMID: ISI:000296999700019.
