# Supplementary material for: Structural Determinants for the Binding of Morphinan Agonists to the μ-Opioid Receptor
Source: PLoS One. 2015 Aug 17;10(8):e0135998. doi: 10.1371/journal.pone.0135998 (PMC4539194; doi:10.1371/journal.pone.0135998)
Supplement: S2 File — (PDF) [file pone.0135998.s006.pdf]

PDB files of morphine, hydromorphone and β-funaltrexamine bound with a lysine residue. The last two columns correspond to the partial charges and atom types (where lowercase letters indicate GAFF types), respectively.

Morphine

```
ATOM      1  C15 MOP     1      -1.125   0.355   1.934 -0.066620 c3
ATOM      2  C16 MOP     1      -2.268  -0.589   1.585 -0.088723 c3
ATOM      3  N1  MOP     1      -3.082  -0.058   0.431 -0.029428 n4
ATOM      4  C17 MOP     1      -4.241  -0.946   0.130 -0.323402 c3
ATOM      5  H17 MOP     1      -4.769  -0.563  -0.730  0.160897 hx
ATOM      6  H18 MOP     1      -4.896  -0.956   0.988  0.160897 hx
ATOM      7  H19 MOP     1      -3.890  -1.946  -0.065  0.160897 hx
ATOM      8  H20 MOP     1      -3.478   0.813   0.747  0.293233 hn
ATOM      9  H15 MOP     1      -2.952  -0.716   2.414  0.115384 hx
ATOM     10  H16 MOP     1      -1.903  -1.559   1.286  0.115384 hx
ATOM     11  H13 MOP     1      -1.513   1.253   2.410  0.060777 hc
ATOM     12  H14 MOP     1      -0.496  -0.144   2.661  0.060777 hc
ATOM     13  C7  MOP     1      -0.313   0.734   0.680  0.074811 c3
ATOM     14  C3  MOP     1       0.279  -0.498   0.056 -0.113238 ca
ATOM     15  C2  MOP     1       1.549  -0.652   0.538  0.237768 ca
ATOM     16  C1  MOP     1       2.324  -1.726   0.146  0.313162 ca
ATOM     17  O2  MOP     1       3.565  -1.850   0.639 -0.574943 oh
ATOM     18  H11 MOP     1       4.024  -2.583   0.249  0.451107 ho
ATOM     19  C6  MOP     1       1.743  -2.610  -0.763 -0.383211 ca
ATOM     20  H3  MOP     1       2.317  -3.457  -1.097  0.219704 ha
ATOM     21  C5  MOP     1       0.474  -2.408  -1.292 -0.175065 ca
ATOM     22  H2  MOP     1       0.105  -3.095  -2.032  0.172469 ha
ATOM     23  C4  MOP     1      -0.288  -1.319  -0.886 -0.005328 ca
ATOM     24  C10 MOP     1      -1.604  -0.882  -1.499 -0.007964 c3
ATOM     25  H5  MOP     1      -2.312  -1.700  -1.579  0.068668 hc
ATOM     26  H6  MOP     1      -1.422  -0.561  -2.520  0.068668 hc
ATOM     27  C9  MOP     1      -2.250   0.329  -0.796 -0.002790 c3
ATOM     28  H4  MOP     1      -2.975   0.771  -1.468  0.087316 hx
ATOM     29  C8  MOP     1      -1.257   1.413  -0.314  0.111395 c3
ATOM     30  H1  MOP     1      -1.848   2.176   0.197  0.033084 hc
ATOM     31  C14 MOP     1      -0.518   2.085  -1.439 -0.351845 c2
ATOM     32  H10 MOP     1      -1.059   2.278  -2.351  0.170786 ha
ATOM     33  C13 MOP     1       0.731   2.485  -1.318 -0.120674 c2
ATOM     34  H9  MOP     1       1.215   2.986  -2.138  0.176227 ha
ATOM     35  C12 MOP     1       1.604   2.337  -0.098  0.280651 c3
ATOM     36  O3  MOP     1       2.822   1.848  -0.568 -0.633473 oh
ATOM     37  H12 MOP     1       3.383   1.608   0.159  0.437959 ho
ATOM     38  H8  MOP     1       1.748   3.334   0.314  0.034773 h1
ATOM     39  C11 MOP     1       1.004   1.454   1.045  0.073973 c3
ATOM     40  H7  MOP     1       0.915   2.039   1.949  0.089080 h1
ATOM     41  O1  MOP     1       1.901   0.385   1.347 -0.353141 os
END
```

Hydromorphone

```
ATOM      1  C11 HMP     1      -2.182  -0.696   1.602 -0.102007 c3
ATOM      2  N1  HMP     1      -3.027  -0.170   0.470 -0.032172 n4
ATOM      3  C17 HMP     1      -4.188  -1.065   0.195 -0.378320 c3
ATOM      4  H17 HMP     1      -3.837  -2.063  -0.004  0.179936 hx
ATOM      5  H18 HMP     1      -4.735  -0.686  -0.655  0.179936 hx
ATOM      6  H19 HMP     1      -4.826  -1.075   1.066  0.179936 hx
ATOM      7  H20 HMP     1      -3.423   0.698   0.798  0.309383 hn
ATOM      8  H8  HMP     1      -2.844  -0.842   2.445  0.131026 hx
ATOM      9  H9  HMP     1      -1.806  -1.658   1.288  0.131026 hx
ATOM     10  C5  HMP     1      -1.050   0.268   1.933 -0.052704 c3
ATOM     11  H4  HMP     1      -0.397  -0.220   2.646  0.068612 hc
ATOM     12  H5  HMP     1      -1.448   1.156   2.419  0.068612 hc
ATOM     13  C1  HMP     1      -0.266   0.669   0.669 -0.024420 c3
ATOM     14  C2  HMP     1       0.351  -0.542   0.027 -0.185342 ca
ATOM     15  C6  HMP     1       1.635  -0.665   0.490  0.325508 ca
ATOM     16  O1  HMP     1       1.973   0.370   1.302 -0.341489 os
ATOM     17  C4  HMP     1       1.050   1.408   1.027  0.046939 c3
ATOM     18  H3  HMP     1       0.969   2.024   1.914  0.132615 h1
ATOM     19  C10 HMP     1       1.560   2.280  -0.131  0.465599  c
ATOM     20  O2  HMP     1       2.713   2.335  -0.395 -0.441300  o
ATOM     21  C15 HMP     1       0.499   3.028  -0.913 -0.001746 c3
ATOM     22  H13 HMP     1       0.002   3.745  -0.261  0.035543 hc
ATOM     23  H14 HMP     1       0.991   3.580  -1.702  0.035543 hc
ATOM     24  C9  HMP     1      -0.531   2.038  -1.478 -0.217214 c3
ATOM     25  H6  HMP     1      -0.027   1.328  -2.124  0.112013 hc
ATOM     26  H7  HMP     1      -1.256   2.567  -2.087  0.112013 hc
ATOM     27  C3  HMP     1      -1.238   1.327  -0.320  0.004780 c3
ATOM     28  H1  HMP     1      -1.821   2.090   0.199  0.043304 hc
ATOM     29  C8  HMP     1      -2.222   0.224  -0.772 -0.052664 c3
ATOM     30  H2  HMP     1      -2.966   0.647  -1.436  0.102963 hx
ATOM     31  C13 HMP     1      -1.571  -0.990  -1.472  0.021194 c3
ATOM     32  H10 HMP     1      -2.256  -1.829  -1.491  0.060800 hc
ATOM     33  H11 HMP     1      -1.451  -0.702  -2.512  0.060800 hc
ATOM     34  C7  HMP     1      -0.218  -1.376  -0.904 -0.005863 ca
ATOM     35  C14 HMP     1       0.563  -2.443  -1.328 -0.164792 ca
ATOM     36  H12 HMP     1       0.198  -3.140  -2.062  0.171593 ha
ATOM     37  C16 HMP     1       1.846  -2.618  -0.818 -0.378638 ca
```

```
ATOM     38  H15 HMP     1       2.432  -3.453  -1.162  0.217873 ha
ATOM     39  C12 HMP     1       2.423  -1.729   0.089  0.298415 ca
ATOM     40  O3  HMP     1       3.663  -1.846   0.577 -0.576910 oh
ATOM     41  H16 HMP     1       4.135  -2.560   0.170  0.459616 ho
END
```

β-funaltrexamine bound with a lysine residue

```
ATOM      1  C14 FNA     1       3.537   1.423  -0.000 -0.054441 ca
ATOM      2  C13 FNA     1       3.160   2.295   1.052  0.023335 c3
ATOM      3  C23 FNA     1       2.107   3.304   0.627  0.049989 c3
ATOM      4  O4  FNA     1       1.729   4.148   1.714 -0.628897 oh
ATOM      5  H34 FNA     1       1.058   4.780   1.414  0.453703 ho
ATOM      6  C24 FNA     1       2.647   4.125  -0.546 -0.312925 c3
ATOM      7  C25 FNA     1       3.913   4.852  -0.179  0.047599 c3
ATOM      8  H37 FNA     1       3.745   5.507   0.649  0.059762 hc
ATOM      9  H38 FNA     1       4.276   5.460  -0.981  0.059762 hc
ATOM     10  H35 FNA     1       1.907   4.841  -0.838  0.162338 hc
ATOM     11  H36 FNA     1       2.868   3.453  -1.349  0.162338 hc
ATOM     12  C12 FNA     1       2.669   1.427   2.226 -0.039475 c3
ATOM     13  H25 FNA     1       3.428   0.730   2.509  0.098965 hc
ATOM     14  H26 FNA     1       2.442   2.031   3.078  0.098965 hc
ATOM     15  C11 FNA     1       1.437   0.688   1.744 -0.133176 c3
ATOM     16  H23 FNA     1       1.716   0.064   0.921  0.100085 hx
ATOM     17  H24 FNA     1       1.064   0.121   2.571  0.100085 hx
ATOM     18  N3  FNA     1       0.374   1.566   1.313  0.021925 n4
ATOM     19  C10 FNA     1      -0.752   0.705   0.870 -0.212710 c3
ATOM     20  C9  FNA     1      -2.072   1.409   0.429 -0.148288 cx
ATOM     21  C7  FNA     1      -2.146   2.382  -0.756 -0.232305 cx
ATOM     22  C8  FNA     1      -2.642   0.957  -0.913 -0.232305 cx
ATOM     23  H17 FNA     1      -2.093   0.265  -1.519  0.150308 hc
ATOM     24  H18 FNA     1      -3.561   0.510  -1.229  0.150308 hc
ATOM     25  H15 FNA     1      -2.844   3.193  -0.740  0.150308 hc
ATOM     26  H16 FNA     1      -1.442   2.999  -1.273  0.150308 hc
ATOM     27  H19 FNA     1      -2.389   1.492   1.448  0.147834 hc
ATOM     28  H20 FNA     1      -0.405   0.127   0.038  0.171814 hx
ATOM     29  H21 FNA     1      -1.022   0.143   1.739  0.171814 hx
ATOM     30  H22 FNA     1       0.003   2.186   2.033  0.332167 hn
ATOM     31  C22 FNA     1       0.967   2.408   0.228 -0.040438 c3
ATOM     32  H33 FNA     1       0.039   2.907   0.043  0.091490 hx
ATOM     33  C21 FNA     1       1.481   1.773  -1.113 -0.085212 c3
ATOM     34  H31 FNA     1       1.628   2.561  -1.822  0.053877 hc
ATOM     35  H32 FNA     1       0.738   1.069  -1.425  0.053877 hc
ATOM     36  C20 FNA     1       2.695   1.056  -0.988  0.092724 ca
ATOM     37  C19 FNA     1       3.126   0.169  -1.952 -0.136750 ca
ATOM     38  H30 FNA     1       2.562  -0.081  -2.777  0.183367 ha
ATOM     39  C18 FNA     1       4.384  -0.357  -1.712 -0.463536 ca
ATOM     40  H29 FNA     1       4.810  -1.002  -2.396  0.289683 ha
ATOM     41  C17 FNA     1       5.079  -0.019  -0.551  0.605046 ca
ATOM     42  O3  FNA     1       6.344  -0.493  -0.409 -0.762200 oh
ATOM     43  H28 FNA     1       6.839  -0.285   0.359  0.471284 ho
ATOM     44  C16 FNA     1       4.836   1.046   0.191 -0.020055 ca
ATOM     45  O2  FNA     1       5.096   1.530   1.435 -0.325941 os
ATOM     46  C15 FNA     1       4.397   2.837   1.359 -0.021906 c3
ATOM     47  H27 FNA     1       4.464   3.487   2.206  0.204935 H1
ATOM     48  C26 FNA     1       4.933   3.716   0.213 -0.001066 c3
ATOM     49  H39 FNA     1       5.054   3.113  -0.662  0.103220 h1
ATOM     50  N4  FNA     1       6.244   4.266   0.600 -0.364572  n
ATOM     51  H40 FNA     1       6.587   4.099   1.508  0.289737 hn
ATOM     52  C27 FNA     1       6.984   4.992  -0.259  0.400043  c
ATOM     53  O5  FNA     1       6.665   5.225  -1.424 -0.452648  o
ATOM     54  C28 FNA     1       8.322   5.499   0.281 -0.000412 c3
ATOM     55  C29 FNA     1       8.661   6.801  -0.465 -0.350893 c3
ATOM     56  C30 FNA     1       7.578   7.884  -0.289  0.829129  c
ATOM     57  O6  FNA     1       7.369   8.704  -1.185 -0.531225  o
ATOM     58  O7  FNA     1       6.894   7.864   0.888 -0.413207 os
ATOM     59  C31 FNA     1       5.925   8.920   0.893 -0.043416 c3
ATOM     60  H44 FNA     1       5.387   8.906   1.818  0.104030 h1
ATOM     61  H45 FNA     1       5.243   8.782   0.081  0.104030 h1
ATOM     62  H46 FNA     1       6.423   9.860   0.785  0.104030 h1
ATOM     63  H42 FNA     1       9.588   7.180  -0.089  0.129991 hc
ATOM     64  H43 FNA     1       8.722   6.571  -1.508  0.129991 hc
ATOM     65  H41 FNA     1       8.278   5.650   1.339  0.101629 hx
ATOM     66  N2  FNA     1       9.376   4.488  -0.092 -0.113705 n4
ATOM     67  H13 FNA     1      10.287   4.946  -0.073  0.317124 hn
ATOM     68  H14 FNA     1       9.149   4.136  -1.022  0.317124 hn
ATOM     69  C6  FNA     1       9.409   3.310   0.845 -0.090484 c3
ATOM     70  H11 FNA     1       8.484   3.281   1.381  0.110937 hx
ATOM     71  H12 FNA     1      10.256   3.451   1.485  0.110937 hx
ATOM     72  C5  FNA     1       9.606   1.949   0.145 -0.047900 c3
ATOM     73  H9  FNA     1       9.446   2.076  -0.906  0.060869 hc
ATOM     74  H10 FNA     1       8.904   1.261   0.569  0.060869 hc
ATOM     75  CG  FNA     1      11.011   1.371   0.363  0.018700 CT
ATOM     76  HG2 FNA     1      11.188   1.302   1.415  0.010300 HC
ATOM     77  HG3 FNA     1      11.699   2.031  -0.124  0.010300 HC
ATOM     78  CB  FNA     1      11.210  -0.028  -0.259 -0.009400 CT
ATOM     79  HB2 FNA     1      12.258  -0.170  -0.422  0.036200 HC
```

```
ATOM     80  HB3 FNA     1      10.628  -0.035  -1.157  0.036200 HC
ATOM     81  CA  FNA     1      10.697  -1.208   0.585 -0.240000 CT
ATOM     82  N   FNA     1      11.221  -1.147   1.949 -0.347900  N
ATOM     83  H   FNA     1      12.235  -1.250   1.929  0.274700  H
ATOM     84  HA  FNA     1       9.631  -1.133   0.643  0.142600 H1
ATOM     85  C   FNA     1      11.041  -2.552  -0.084  0.734101  C
ATOM     86  O   FNA     1      10.214  -3.128  -0.798 -0.589401  O
END
```
