# Supplementary material for: Structural Determinants for the Binding of Morphinan Agonists to the μ-Opioid Receptor
Source: PLoS One. 2015 Aug 17;10(8):e0135998. doi: 10.1371/journal.pone.0135998 (PMC4539194; doi:10.1371/journal.pone.0135998)
Supplement: S3 File — (PDF) [file pone.0135998.s007.pdf]

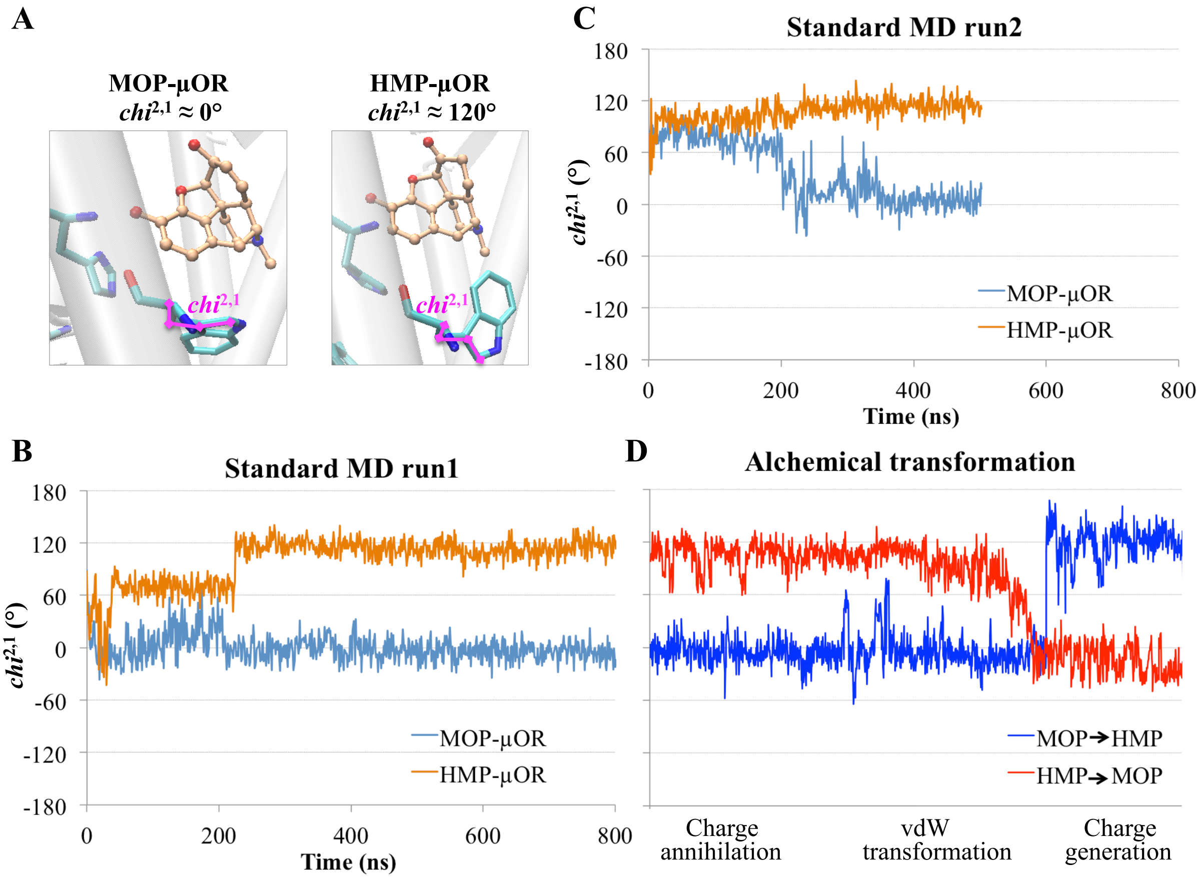

Fig. S7-1. Simulations of MOP-µOR and HMP-µOR: the W293 side chain orientation. (A) Basically, the W293 dihedral angle $chi^{2,1}$ fluctuates around two favorable values (~0˚ in MOP-µOR and ~120˚ in HMP-µOR). The $chi^{2,1}$ value is plotted as a function of simulation time in **(B, C)** the two independent MD simulations for each system and in **(D)** the forward and backward alchemical free energy perturbations in the bound state.

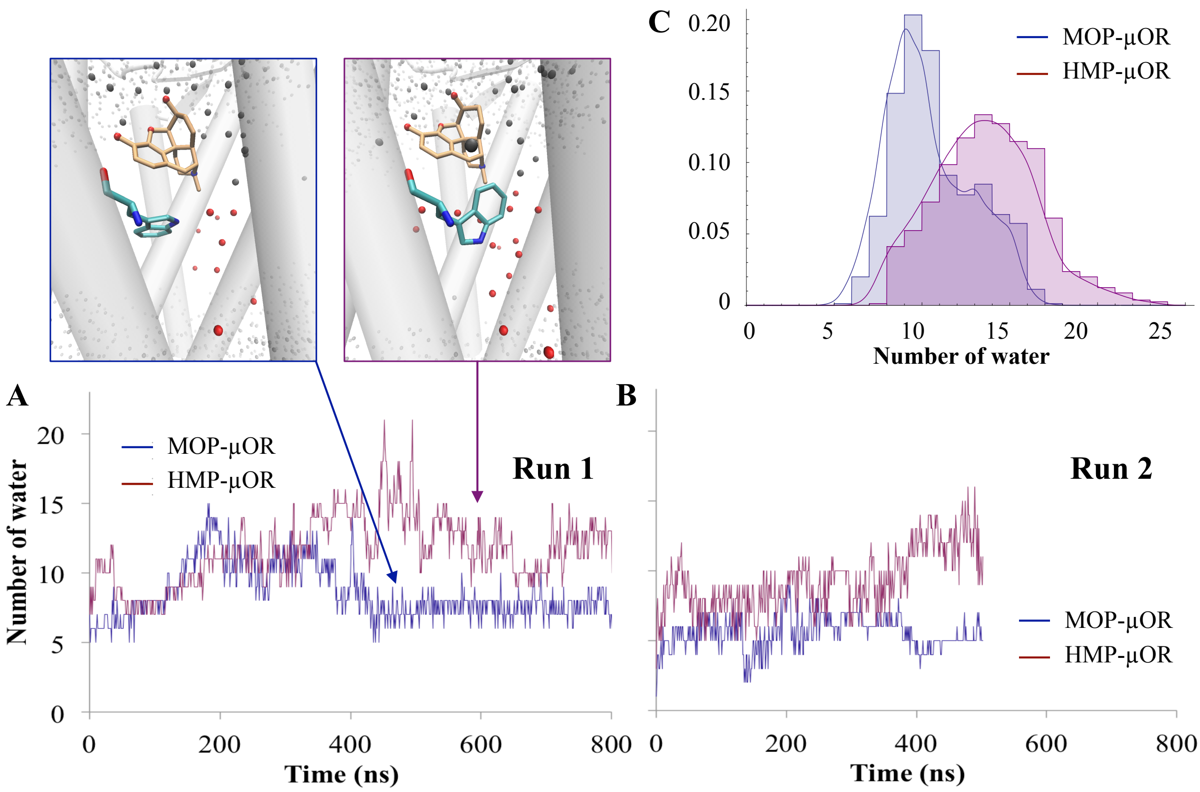

Fig. S7-2. MD simulations of MOP-µOR and HMP-µOR: number of water molecules inside the µOR binding cavity. (A, B) The number plotted as a function of time in the two independent MD simulations. **(C)** Normalized histograms of the data plotted in **(A)**.

We performed Welch's *t*-test on the two sets of data plotted in Fig. S7-2A. The results (Table S7-1) indicate that the difference shown in Fig. S7-2A is statistically significant.

Table S7-1. Welch's *t*-test [a] on the data shown in Fig. S7-2A.

P-value [b]	Difference in mean values	Standard error	95% confidence interval of the difference	Intermediate values used in the test	
				t	d*f*
< 0.0001	3.086	0.133	2.825–3.347	23.2261	1541

[a] The test assumes normal distribution of the two datasets. Although this is not the case for MOP, the test has been shown to be still robust with a large sample size [1] (i.e. ≥200 [2]).
[b] P-value much less than the threshold (0.05) indicates significant difference between the two datasets.

Table S7-2. Distances (Å) between the agonists and selected protein residues averaged over two independent MD runs.

	MD run	I296	M151	G325	I322
MOP	Run 1 (0.8 µs)	2.4 ± 0.2	2.2 ± 0.2	2.4 ± 0.3	3.3 ± 0.5
	Run 2 (0.5 µs)	2.4 ± 0.2	2.2 ± 0.2	2.9 ± 0.7	2.3 ± 0.2
HMP	Run 1 (0.8 µs)	2.4 ± 0.2	2.3 ± 0.2	5.0 ± 0.3	2.7 ± 0.2
	Run 2 (0.5 µs)	2.6 ± 0.3	2.1 ± 0.2	5.8 ± 0.4	2.6 ± 0.3

Supporting references

1. Fagerland MW. t-tests, non-parametric tests, and large studies--a paradox of statistical practice? BMC medical research methodology. 2012;12:78. Epub 2012/06/16. doi: 10.1186/1471-2288-12-78. PubMed PMID: 22697476; PubMed Central PMCID: PMC3445820.
2. Fagerland MW, Sandvik L. Performance of five two-sample location tests for skewed distributions with unequal variances. Contemp Clin Trials. 2009;30(5):490-6. doi: Doi 10.1016/J.Cct.2009.06.007. PubMed PMID: ISI:000270073800018.
